# The Role of Drug Repurposing in the Development of Novel Antimicrobial Drugs: Non-Antibiotic Pharmacological Agents as Quorum Sensing-Inhibitors

**DOI:** 10.3390/antibiotics8040270

**Published:** 2019-12-17

**Authors:** Márió Gajdács, Gabriella Spengler

**Affiliations:** 1 Department of Medical Microbiology and Immunobiology, Faculty of Medicine, University of Szeged, Dóm tér 10, 6720 Szeged, Hungary; 2 Department of Pharmacodynamics and Biopharmacy, Faculty of Pharmacy, University of Szeged, Eötvös utca 6, 6720 Szeged, Hungary; spengler.gabriella@med.u-szeged.hu

**Keywords:** drug repurposing, non-antibiotics, antimicrobials, pharmaceuticals, quorum sensing, quorum quenching, screening

## Abstract

*Background:* The emergence of multidrug-resistant organisms (MDROs) is a global public health issue, severely hindering clinicians in administering appropriate antimicrobial therapy. Drug repurposing is a drug development strategy, during which new pharmacological applications are identified for already approved drugs. From the viewpoint of the development of virulence inhibitors, inhibition of quorum sensing (QS) is a promising route because various important features in bacterial physiology and virulence are mediated by QS-dependent gene expression. *Methods:* Forty-five pharmacological agents, encompassing a wide variety of different chemical structures and mechanisms of action, were tested during our experiments. The antibacterial activity of the compounds was tested using the broth microdilution method. Screening and semi-quantitative assessment of QS-inhibition by the compounds was performed using QS-signal molecule-producing and indicator strains. *Results:* Fourteen pharmaceutical agents showed antibacterial activity in the tested concentration range, while eight drugs (namely 5-fluorouracil, metamizole-sodium, cisplatin, methotrexate, bleomycin, promethazine, chlorpromazine, and thioridazine) showed dose-dependent QS-inhibitory activity in the *in vitro* model systems applied during the experiments. *Conclusions:* Virulence inhibitors represent an attractive alternative strategy to combat bacterial pathogens more efficiently. Some of the tested compounds could be considered potential QS-inhibitory agents, warranting further experiments involving additional model systems to establish the extent of their efficacy.

## 1. Introduction

The introduction of antibiotics into clinical medicine was one of the main prerequisites for the development of our present-day healthcare; previously deadly infections have suddenly become treatable, and the introduction of various medical interventions (invasive surgery, organ transplantation) and specialties (oncological care, neonatology) became possible [1,2]. The emergence of multidrug-resistant organisms (MDROs) is a global public health issue, which severely hinders clinicians in defining appropriate patient treatment options and treatment regimens [3,4]; the ramifications of the spread of drug-resistant pathogens are biggest in developing countries, significantly contributing to morbidity and mortality [5,6]. Based on their overall impact (mortality-wise and economically), the group of “ESKAPE” pathogens (this acronym was first proposed by Louis B. Rice in 2008), namely **E:** *Enterococcus faecium*, **S:** *Staphylococcus aureus* or recently *Stenotrophomonas maltophilia*, **K**: *Klebsiella pneumoniae* or recently **C**: *Clostridioides difficile*, **A**: *Acinetobacter baumannii*, **P**: *Pseudomonas aeruginosa*, **E**: *Enterobacter* spp., or recently *Enterobacteriaceae*) present the most clinical challenges [7]. Some of these bacteria have also been included in the priority pathogens list, which was developed for pharmaceutical companies by the World Health Organization (WHO) to guide the development of new antimicrobial drugs [8,9,10]. Some of the main public health authorities (e.g., the WHO, the European Center for Disease Prevention and Control (ECDC), and the Centers for Disease Control and Prevention in the US (CDC)) have all published reports on the global impact of bacterial drug resistance and urged for global action to be taken [11,12,13,14]; among these publications; however, the O’Neill report (sequestered by the National Health Service (NHS) of the United Kingdom) is probably the most pessimistic, projecting 10 million deaths per year by 2050 and 100 billion USD worth of economic burden [15]. In addition to their inappropriate use in animal husbandry and human medicine, one of the main concerns regarding the therapy of MDROs is the unavailability of novel antibacterial agents [16,17,18]. The absolute costs of antimicrobial research and development (R&D), the financial risks associated with the organization of clinical trials, the inevitable and rapid emergence of drug-resistant mutants against these new drugs and the comparatively modest return on investment (ROI; which is a performance measure used to evaluate the efficiency of an investment) has resulted in pharmaceutical companies shifting their interests towards the therapy of chronic illnesses or leaving behind the antimicrobial research programs entirely [19,20,21,22]. This has resulted in a ‘dry’ antibiotic pipeline and no new broad-spectrum antibiotics since the introduction of the fluoroquinolones in the 1980s [23,24].

In lieu of new antibiotics, novel strategies have been proposed to combat bacterial pathogens more efficiently—among other things, combination therapy (the use of two or more existing antibiotics simultaneously) and the use of adjuvants (non-antibiotic drugs co-administered with an existing antibiotic) all present possible alternatives [25,26]. These adjuvants include agents already used in clinical practice, such as monoclonal antibodies (e.g., bezlotoxumab, neutralizing *Clostridioides difficile* toxin B) [27], β-lactamase inhibitors (e.g., clavulanic acid, avibactam) [28], and others, such as efflux pump inhibitors (EPIs; compounds capable of inhibiting bacterial transporter proteins that utilize proton motive force or the hydrolysis of ATP to remove various chemicals from bacterial cells) [29,30], modulators of bacterial membrane potential and membrane permeabilizers [26]. However, it’s worth noting that at present, none of the abovementioned EPIs or membrane permeabilizers can be used in clinical practice, due to the very high concentrations required for them to be effective, which usually corresponds to debilitating toxicity in vivo [29,30]. Another promising approach to treat bacterial infections is through the use of virulence inhibitors (or ‘pathoblockers’) [31]. The rationale behind the use of these compounds is that they do not affect the viability of bacterial cells in vivo; instead, they inhibit the synthesis or expression of bacterial virulence factors (e.g., exotoxins, secreted bacterial enzymes, biofilm) which are key in their pathogenesis, or modulate their genetic plasticity (i.e., competence) [32,33,34,35]. The potential advantage of these agents (compared to antibiotics) is that the selection pressure exerted by these drugs (and consequently, the chance of resistance development) is expected to be much lower; therefore, the rapid emergence of drug-resistant mutants is unlikely [36,37]. Some reports also suggest that anti-virulence drugs may have minor effects on the gut microbiome: they should be able to exert their activity without causing ‘collateral damage’ [38,39].

Bacterial quorum sensing (QS) is a distinct mechanism of cell-cell communication, during which bacteria can ‘sense’ the density of cells in the surrounding environment, resulting in the expression or suppression of specific genes [40,41]. Surrounding bacterial cell population density is established by the detection of diffusible signal molecules produced by surrounding cells, in addition, self-produced signals are also detected (activating positive feed-back circuits); if the concentration of these signal molecules (or autoinducers) reaches a critical concentration, transcription changes occur in various genes, which are important for benefits in fitness and reproductive success in their specific niche [40,42,43]. The phenomenon of QS was first described in 1968 by Kempner and Hanson in *Vibrio fischeri* (postulating that the culture media contained a luminescence inhibitor, which was removed if large numbers of bacteria were present [44]); however, the true mechanism of QS (namely, the initiation of phenotypic changes by the accumulation of autoinducers secreted by bacteria) was reported by Nealson et al. in 1970 [45], and Eberhard et al. in 1972 [46]. QS-signal molecules encompass a wide variety of structurally different molecules: in Gram-positive bacteria, peptide-based signal molecules (AIPs, autoinducing peptides) are most frequently detected, while in Gram-negatives, acyl-homoserine lactone-derivatives (AHLs) are the most prevalent; interestingly, some signal molecule-types (e.g., AI-2, a derivative of dihydroxy-2,3-pentanedione) may be detected by a wide range of bacteria, while others (e.g., *Pseudomonas* quinolone signal (PQS), diffusible signal factor (DSF)) are specific to one or a very few species [40,41,42,43,47,48]. The elimination or inhibition of QS-signal transmission is termed quorum quenching (QQ), which may be mediated by the use of signal-antagonists, inhibition of signal sensing, or synthesis, influencing bacteria on the level of gene expression and by the degradation of these signal molecules [42,47]. Synthetic molecules (i.e., quorum quenching based on inhibition) may inhibit signal transduction mechanisms relevant in virulence factor-expression of relevant pathogens, therefore disarming them in vivo [41,49,50]. From the viewpoint of the development of virulence inhibitors, quorum quenching is a promising route, because various important bacterial features in physiology and virulence (e.g., production of toxic shock syndrome toxin in *Staphylococcus aureus*, elastase in *P. aeruginosa*, protease in *V. cholerae*; activity of bacterial secretion systems (e.g., *Salmonella* species) and efflux pumps (e.g., *P. aeruginosa, Escherichia coli*), biofilm-production (e.g., *P. aeruginosa*, *Acinetobacter baumannii*, *Stenotrophomonas maltophilia*); induction of bacterial competence (e.g., *Streptococcus pneumoniae*), motility (e.g., *P. aeruginosa*), adhesion (e.g., *E. coli, Klebsiella pneumoniae*) and pigment-production (e.g., *Chromobacterium violaceum*, *Serratia marcescens*, *P. aeruginosa*)) are mediated by QS-dependent gene expression [31,40,51,52]. Due to its promise for future applications in human medicine, research on quorum sensing and quorum quenching has garnered significant attention in the last 15–20 years (see Supplementary Figure S1 for bibliometric assessment).

Drug repurposing (also called drug re-profiling or repositioning) is a drug development strategy, during which new pharmacological uses are identified for already approved drugs, outside of their original designated medical indications [39,53]. This strategy offers various advantages: the chemical and technological aspect of these molecules are already established, the toxicity, safety and pharmacokinetic profile of the drug is known; therefore, early stages of the drug development process (preclinical *in vitro* and animal models, Phase I–II clinical trials) may be avoided, leading to substantial savings for the pharmaceutical companies [39,53,54]. Although the costs of organizing Phase III–IV trials are still considerable, if the new indication for the drugs is appropriate, drug companies may still expect sizeable ROIs. Previously, drug repurposing was mainly based on serendipitous discoveries or retrospective analyses of clinical data; nowadays, there are initiatives to systematically screen the existing drug pool for off-target effects, which may be suitable for the development of additional clinical indications [39,53,54]. Examples of the success of drug repurposing include the use of thalidomide (morning sickness → multiple myeloma, erythema nodosum leprosum), minoxidil (hypertension → alopecia), ketoconazole (antifungal drug → Cushing syndrome), aspirin (analgesia → colorectal cancer), and sildenafil (angina pectoris → erectile dysfunction → pulmonary hypertension) in new clinical indications [53].

Drug repurposing is also a promising strategy in the therapy of bacterial infections: many pharmaceuticals have secondary mechanisms of action (some of which are not fully characterized), which allows them to be efficacious against various pathogens, either as directly acting antibacterial agents or as virulence inhibitors [55]. For this reason, there is interest in the screening of the existing pool of pharmaceutical agents as anti-virulence drugs; however, there are significant gaps in knowledge in this field [56,57,58]. The aim of our present study was to assess the suitability of a selection of non-antibiotic pharmacological agents as QS-inhibitors, with various *in vitro* bacterial model systems, using disk-diffusion based QS-inhibitory (DDBQSI) assays.

## 2. Results and Discussion

### 2.1. Antibacterial Activity

Among the tested pharmaceutical agents, fourteen (namely *celecoxib, mebendazole, ivermectin, verapamil, promethazine, chlorpromazine, thioridazine, methotrexate, doxorubicin, bleomycin, atorvastatin, simvastatin, clotrimazole*, and *fluconazole*) showed antibacterial activity in the tested concentration range (0.25–250 µg/mL), while the other agents in the study had no relevant antibacterial properties on the bacterial strains used in this study (minimal inhibitory concentrations [MIC] > 250 µg/mL; Table 1). The most potent antibacterial activity was noted for chlorpromazine, thioridazine and mebendazole (consistently for all tested strains), in addition, MICs were recorded in the moderate range regarding the tested statins (for *Enterobacter cloacae*), promethazine (for *Chromobacterium violaceum* wt85 and CV026), celecoxib (for *S. aureus*) and ivermectin (for *S. aureus*). The antibacterial activity of the tested antipsychotic drugs became more potent with the progression of the different generation drugs (promethazine is a non-selective, first-generation phenothiazine, while thioridazine is a newer drug of the same group) [59]; these compounds have been extensively characterized as efflux pump inhibitors, while the antibacterial activity of these drugs is partly attributed to their intercalation into DNA [60]. Anthracyclines (including doxorubicin) and bleomycin are frequently termed ‘*anticancer antibiotics*’; therefore, it is not surprising that the antibacterial properties of these drugs were observed [61,62]. Similarly to the phenothiazines, their antibacterial activity is also attributed to bacterial DNA-intercalation, while it is debated whether their potency to produce semiquinone-based oxidative free radicals in the presence of Fe^2+^-ions (which is an important factor of their anticancer activity in vivo) is important in this regard [61,62,63,64]. Atorvastatin was effective in a 1–2-fold lower dose than simvastatin; although the exact mechanism of action is uncertain, some reports suggest that they interfere with the mevalonate pathway (similarly to eukaryotic cells) and the synthesis of the major lipid constituents of cell membrane microdomains [51,65,66]. In line with previous reports, ivermectin, celecoxib, and the azole-type antifungal agents were only effective against Gram-positive bacteria (in our case S. aureus ATCC 25923) [67,68,69]. The MIC values recorded in this experiment were used to set the starting doses of these drugs in the QS-inhibition assays to distinguish between their quorum quenching and antibacterial properties [70]. The MICs of acridine orange have been previously reported at 125 µg/mL on the QS-sensor strains (*C. violaceum* wt85, CV026, and *Serratia marcescens* AS-1) and >250 µg/mL for the other tested bacterial strains [71]. The results on the control strains (*S. aureus* and *E. coli* ATCC strains) were adequate and in line with findings in the literature [59,60,65,67,68,69].

### 2.2. Screening and Semi-Quantitative Assessment of QS-Inhibitory Activity

**Figure 1:**
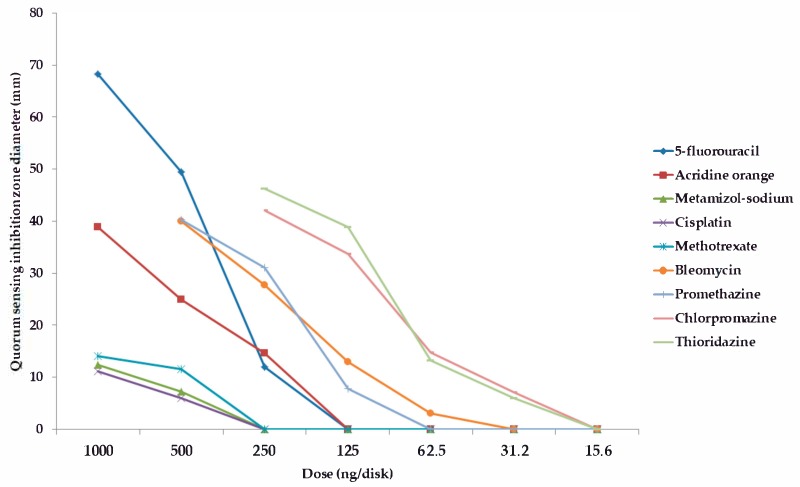
Dose-dependent quorum sensing-inhibitory activity of tested compounds. Model system: *C*. *violaceum* CV026, and *E*. *cloacae* 31298 (quorum sensing inhibition zone diameters with SD values are in the Supplementary Material Table S1).

**Figure 2:**
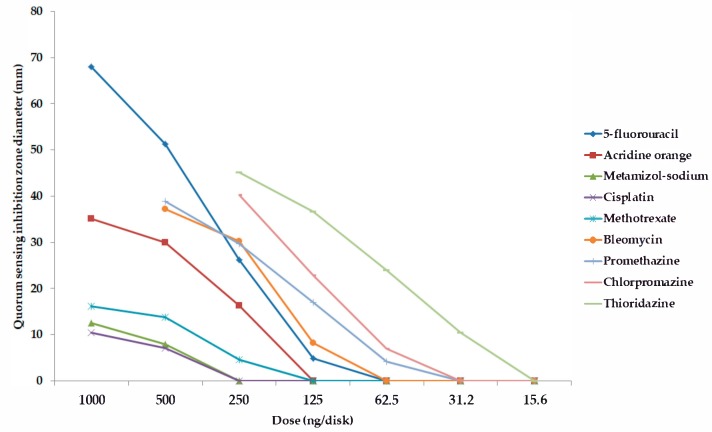
Dose-dependent quorum sensing-inhibitory activity of tested compounds. Model system: *C*. *violaceum* CV026, and *S*. *paucimobilis* Ezf 10–17 (quorum sensing inhibition zone diameters with SD values are in the Supplementary Material Table S1).

**Figure 3:**
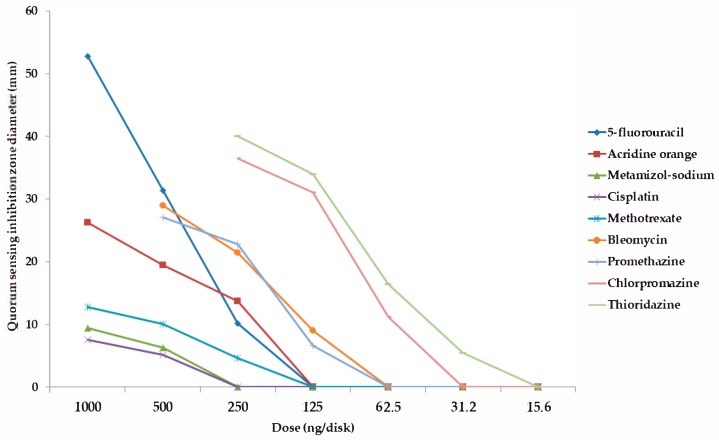
Dose-dependent quorum sensing-inhibitory activity of tested compounds. Model system: *C*. *violaceum* CV026, and *Novosphingobium* spp. Rr 2–17 (quorum sensing inhibition zone diameters with SD values are in the Supplementary Material Table S1).

**Figure 4:**
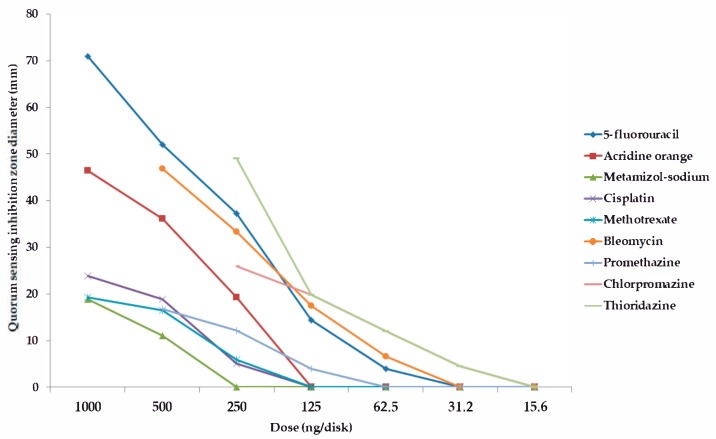
Dose-dependent QS-inhibitory activity of tested compounds. Model system: *S*. *marcescens* AS-1. (quorum sensing inhibition zone diameters with SD values are in the Supplementary Material Table S1).

Among the forty-five tested pharmaceutical compounds, eight drugs (namely 5-*fluorouracil, metamizole-sodium, cisplatin, methotrexate, bleomycin, promethazine, chlorpromazine,* and *thioridazine*) showed relevant QS-inhibitory activity in the cross-inoculation experiments with *C. violaceum* wt85 and *S*. *marcescens* AS-1; therefore, these compounds were included in the parallel inoculation assay (with *S*. *marcescens* AS-1, and the *C. violaceum* CV026 + AHL-producer-pair combinations) to quantify the QS-inhibition of these agents, while the other pharmaceutical agents were not tested further; the result of these experiments is presented in Figure 1, Figure 2, Figure 3 and Figure 4 and in the Supplementary Table S1 (expressed as QS-inhibition zone diameters with SD values for each respective model systems). DMSO, 70% ethanol, 85% glycerol, and acetone were also tested as solvent controls in the cross-inoculation experiments, where they presented with no QS-inhibitory effects.

Compared to acridine orange (AO), the QS-inhibitory effects of metamizole-sodium, cisplatin and methotrexate were less potent, with 2–10 times smaller QS-inhibition zones), while the phenothiazine-derivatives, bleomycin, and 5-fluorouracil exhibited concentration-dependent QS-inhibitory activity, which was more potent than AO in almost all cases when tested at the same or even at lower doses (QS-inhibition zones 1.5–3-times larger than in the case of AO, or the activity in doses where the positive control showed no QS-inhibition; Figure 1, Figure 2, Figure 3 and Figure 4). When it comes to the phenothiazines, the potency of QS-inhibition increased with the progression of the different generation drugs (promethazine → thioridazine), similarly to their antibacterial activity. Interestingly, some agents, which presented with antibacterial activities (*celecoxib, mebendazole, ivermectin, verapamil, doxorubicin, atorvastatin, simvastatin, clotrimazole, fluconazole*) did not show QS-inhibitory properties, while others (metamizole-sodium, cisplatin) had no antibacterial activity while being QS-inhibitors (Figure 1, Figure 2, Figure 3 and Figure 4). Overall, the most potent QS-inhibitor (among the tested pharmaceutical compounds) was 5-fluorouracil, and the *S*. *marcescens* model system presented as the most sensitive for the QS-inhibitory activity of the tested compounds (i.e., inhibition of prodigiosin pigment production), while among the *C*. *violaceum* CV026 and AHL-producer pair system, the following order may be set up in regards to sensitivity, based on our results: 1. *C*. *violaceum* CV026 and *Sphingomonas paucimobilis* Ezf 10–17 (most sensitive), 2. *C*. *violaceum* CV026 and *E*. *cloacae* 31298, 3. *C*. *violaceum* CV026 and *Novosphingobium* spp. Rr 2–17 (least sensitive) (Figure 1, Figure 2, Figure 3 and Figure 4.). The mechanism of action of the active pharmaceutical drugs has not been described: as signal molecule-degradation (i.e., quorum quenching by degradation) mainly occurs with compounds with enzymatic activity (e.g., the human paraoxonase enzyme, which is essentially a lactonase), findings of other studies in the literature suggest that these drugs exert their QS-inhibitory properties through inhibition of signal detection or through modifying gene expression in these bacteria [42,47].

There are several studies reporting on the QS-inhibitory activity of antibiotics (e.g., azithromycin, gentamicin, tobramycin, and fluoroquinolones) or the successful use of QS-inhibitory compounds together with conventional antibiotics *in vitro* and in animal models [72,73,74]. Non-antibiotic pharmaceutical drugs and compounds derived from various natural sources and foos have attracted reasonable attention as novel antibacterial agents or adjuvant compounds (efflux pump inhibitors, membrane permeabilizers, QS-inhibitors) because their physicochemical and *in vivo* biological properties have been previously described [25,26,30,39,53], a summary of relevant compounds and corresponding literature is presented in Table 2. The characterization of the antibacterial and QS-inhibitory activity of the abovementioned compounds was performed through the utilization of different *in vitro* model systems (e.g., *Aeromonas* spp., *Agrobacterium tumefaciens*, *Bacillus subtilis*, *Burkholderia cepacia*, *Chromobacterium* spp., *E. coli*, *Serratia* spp., *P. aeruginosa* PAO1, *V. harveyi*, *S. aureus*, *S. maltophilia*) and molecular testing methods (e.g., detection of differences in gene expression levels with PCR) [74,75,76,77,78,79,80,81,82,83].

Although it is difficult to assess the detected activity of these drug molecules in relation to previous publications, because most of these reports used different model systems (e.g., inhibition of swarming motility or elastase-, protease-production of *P. aeruginosa* PAO1, to the detection of gene-expression changes in *P. aeruginosa*, *S. maltophilia* or *S. aureus*, or experimental animal-based systems, where a lung, soft tissue, or other infection models are used), it can be observed that most of the mentioned compounds exerted QS-inhibitory properties in the same dose range or in doses 1–2-fold lower than in our studies, these differences are presumably due to the different sensitivities of these model systems utilized, while in animal systems, the tissue distribution of the compounds should also be taken into consideration [65,66,67,68,69,70,71,72,73,74,75,76,77,78,79,80,81,82,83].

Some reports also suggested that various vitamins have potent adjuvant properties, enhancing the bactericidal activity of antibiotics (Table 2), and they also exert their own antibacterial activities in very high concentrations [103,104,105,106,107]. Nevertheless, the tested vitamins and antioxidants (Vitamin B_1_, Vitamin B_6_, Vitamin B_12_, Vitamin C, Vitamin D, Vitamin E, Coenzyme Q_10_) did not show any antibacterial or QS-inhibitory activity during our experiments.

***Highlights of the study***: during our experiments, the antibacterial and QS-inhibitory capacities of 45 currently used pharmacological agents (sourced from diverse clinical indications and molecular characteristics) have been tested in a semi-quantitative *in vitro* model, for which such data were not available whatsoever, or data were generated on different model systems. *Fourteen* of the tested drugs showed varying degrees of antibacterial activity on the tested bacterial strains, while *five* drugs (promethazine, chlorpromazine, thioridazine, 5-fluorouracil, and bleomycin) showed dose-dependent QS-inhibitory activity, which was more potent than acridine orange. Based on the results of this experiment, the characterization of active pharmaceutical compounds on different QS-based model systems is highly recommended, in addition, the continuous screening of the existing drug pool may result in the establishment of a library of clinically relevant drugs with virulence-modulating properties. The *in vitro* antibacterial properties of the tested drugs highlight the tendency of some non-antibiotic pharmacological agents to affect bacterial viability, which is important in the context of the human gut microbiome: in addition to antibiotics, these drugs may also have detrimental effects on the species composition of gut bacteria (leading to disease), therefore this study provides novel insights in this aspect as well [118].

***Limitations of the study:*** during our experiments, the QS-inhibitory activity of the compounds was only tested against strains, where cell-cell communication is based on AHL-signal molecules and their activity was not characterized on the genetic level with molecular methods. To further establish the QS-inhibitory and anti-virulence properties of the tested drugs, further experiments with additional Gram-positive (e.g., including AI-2-producing *Bacillus* species, toxin-producing *S. aureus*), and Gram-negative (e.g., elastase-production and motility-assays with *P. aeruginosa* (PQS-mediated), *S. maltophilia* (DSF-mediated) bacterial model systems should be considered.

## 3. Materials and Methods

### 3.1. Chemicals

#### 3.1.1. Pharmaceutical Compounds

Forty-five pharmacological agents, encompassing a wide variety of different chemical structures and mechanisms of action were tested during our experiments: ***1. non-steroidal anti-inflammatory drugs (NSAIDs):*** acetylsalicylic acid (Sigma-Aldrich; Budapest, Hungary; will be listed as SA in the subsequent text), indomethacin (Sanofi; Paris, France; will be listed as SP in the subsequent text), metamizole-sodium (SF), diclofenac (SA), celecoxib (Pfizer Hungary Ltd.; Budapest, Hungary), acetaminophen (SA), ***2. antiviral and antifungal drugs:*** acyclovir (Teva Pharmaceuticals; Petah Tikva, Israel; will be listed as TPh in the subsequent text), cidofovir (SA), amantadine (SA), clotrimazole (TPh), fluconazole (SA), terbinafine (GlaxoSmithKline Hungary Ltd., Budapest, Hungary), ***3. anthelmintic drugs:*** mebendazole (Richter Pharmaceuticals; Budapest, Hungary; will be listed as RPh in the subsequent text), ivermectin (SA), ***4. anti-allergy medications (H_1_-receptor antagonists and decongestants):*** cetirizine (SA), azelastine (SA), xylomethazoline (SA), ***5. drugs targeting the cardiovascular system:*** metoprolol succinate (SA), enalapril maleate (SA), valsartan (SA), verapamil (TPh), simvastatin (SA), atorvastatin (SA), ***6. mucolytics and antitussives:*** ambroxol (TPh), acetyl-cysteine (TPh), guaifenesin (SA), ***7. neuroleptic drugs:*** promethazine (SA), chlorpromazine (SA), thioridazine (SA), ***8. antimetabolite anticancer agents:*** methotrexate (Ebewe Pharma, Unterach am Attersee, Austria), 5-fluorouracil (TPh), gemcitabine (TPh), ***9. alkylating anticancer agents:*** cyclophosphamide (Baxter; Deerfield, IL, United States), cisplatin (TPh), ***10. anticancer drugs affecting the microtubule system or topoisomerase-enzymes:*** vincristine (TPh), paclitaxel (TPh), doxorubicin (TPh), topotecan (SA), bleomycin (TPh), ***11. vitamins and antioxidants:*** Vitamin B_1_ (EGIS Pharmaceuticals; Budapest, Hungary; will be listed as EGIS in the subsequent text), Vitamin B_6_ (EGIS), Vitamin B_12_ (RPh), Vitamin C (SA), Vitamin D (EGIS), Vitamin E (SA), Coenzyme Q_10_ (SA). Pharmaceutical compounds were dissolved in phosphate-buffered saline, with the exception of simvastatin and atorvastatin, which were dissolved in DMSO, in addition to Vitamin D and Coenzyme Q_10_, which were dissolved in acetone and 70% ethanol, respectively.

#### 3.1.2. Media Constituents and Other Reagents

Bacteriological agar (Bio-Rad Hungary Ltd.; Budapest, Hungary), tryptone (Thermo Fischer Scientific; Waltham, MA, USA; will be listed as TFS in the subsequent text), yeast extract (TFS), d-glucose (SA), kanamycin (SA), NaCl (SA), K_2_HPO_4_ (SA), KH_2_PO_4_ (SA), MgSO_4_ × 7H_2_O (SA), CaCl_2_ × 2H_2_O (SA), FeSO_4_ × 7H_2_O (SA), Na_2_EDTA (SA), MnSO_4_ × 7H_2_O (SA), ZnSO_4_ × 7H_2_O (SA), Na_2_MoO_4_ × 2H_2_O (SA), CoCl_2_ × 6H_2_O (SA), dimethyl-sulfoxide (DMSO; SA), acetone (SA), acridine orange (AO; SA), 70% ethanol (SA), 85% glycerol (SA), and phosphate-buffered saline (PBS; SA). During the preparation of the modified Luria-Bertani agar (LB*-A, Bio-Rad Hungary Ltd.; Budapest, Hungary), the following stock solutions were used: 5% Fe-EDTA stock solution, 3% CaCl_2_ stock solution, and a microelement stock solution (containing 1.0 g MnSO_4_ × 7H_2_O, 0.5 g ZnSO_4_ × 7H_2_O, 25 mg Na_2_MoO_4_ × 2H_2_O and 2.5 mg CoCl_2_ × 6H_2_O per 100 mL). The stock solutions were aliquoted in 50 mL centrifuge tubes and kept at –20 °C till use.

### 3.2. Bacterial Strains

The following bacterial strains were used during our QS-inhibition experiments: *Chromobacterium violaceum wt85* (wild-type strain, characterized by the AHL signal molecule-mediated production of the purple violacein pigment, capable of the production of endogenous QS-signal molecule (N-hexanoyl-L-HSL)), *C. violaceum CV026* (Tn5 transposase-mutant, AHL-signal molecule indicator strain: incapable of endogenous QS-signal molecule-production, but produces purple violacein pigment in the presence of external AHL stimuli) [70,71], *Enterobacter cloacae* clinical isolate no. 31298 (isolated from a wound sample, AHL-producing-strain (used with *C. violaceum* CV026)) [70,71], *Sphingomonas paucimobilis Ezf 10–*17 (isolated from a tumor of the “Ezertűfű” variety of the common grapevine (*Vitis vinifera*), AHL-producing-strain (used with *C. violaceum* CV026)), *Novosphingobium* spp. Rr 2–17 (isolated from a tumor of the “Rajnai rizling” variety of the common grapevine (*Vitis vinifera*), AHL-producing-strain (used with *C. violaceum* CV026)) [119], *Serratia marcescens* AS-1 (characterized by the production AHL signal molecule-mediated production of the orange-red pigment prodigiosin (2-methyl-3-pentyl-6-methoxyprodigiosin), capable of endogenous QS-signal molecule-production (N-hexanoyl-L-HSL)) [120]. In addition, *Staphylococcus aureus* ATCC 25923 and *Escherhichia coli* ATCC 25922 were used as control strains.

The QS-sensory and indicator of bacterial strains for our experiments were kindly provided by Dr. Ernő Szegedi (Institute of Viticulture and Enology, National Agricultural Research Center; Badacsonytomaj, Hungary). For shorter time periods (<1 month), the bacterial strains were maintained on Luria-Bertani (LB) agar, while for longer periods, the strains were kept in a –80 °C freezer, in a 1:4 mixture of 85% glycerol and liquid Luria-Bertani (LB-B) medium. For the maintenance purposes of *C. violaceum* CV026, the media were also supplemented with kanamycin.

### 3.3. Culture Media

The following culture media were used during our experiments: cation-adjusted Mueller–Hinton broth (CMH-B; Bio-Rad Hungary Ltd., Budapest, Hungary), Luria–Bertani broth (LB-B), and Luria–Bertani agar (LB-A) (Bio-Rad Hungary Ltd., Budapest, Hungary) which were purchased, while the modified Luria–Bertani agar (LB*-A) (which was used during the QS-inhibition assays; containing 8.0 g tryptone, 5.0 g yeast extract, 5.0 g NaCl, 2.0 g d-glucose, 1.0 g K_2_HPO_4_, 0.2 g MgSO_4_ × 7H_2_O, 10 mL 3% CaCl_2_ stock solution, 5 mL Fe-EDTA stock solution, 1 mL microelement stock solution, and 12.0 g of bacteriological agar per 1 L of media; pH was adjusted to 7.0–7.2) was prepared in-house [71].

### 3.4. Antibacterial Activity

As a part of our study, the antibacterial activity of non-antibiotic pharmaceutical compounds on QS-sensory and signal molecule-producing bacterial strains was determined. The purpose of the assay was to screen for possible antibacterial activity of the tested compounds so that later on, their potential QS-inhibitory activities and their bacterial population density-reducing properties (due to their bacteriostatic or bactericidal effects) in the subsequent QS-inhibition assays could be distinguished. The minimum inhibitory concentrations (MICs) of the tested compounds were determined using the standard broth microdilution (BMD) method, based on the recommendations of the Clinical and Laboratory Standards Institute (CLSI; M07-A10). The experiments were performed in 96-well polystyrene microtiter plates, using cation-adjusted Mueller–Hinton broth (CMH-B), the tested concentrations of the compounds were ranging between 0.25–250 µg/mL. During the experiments with *S. aureus* ATCC 25922, CMH-B was supplemented by 2% NaCl. The plates were incubated at 37 °C in an air thermostat; the MIC values of the tested compounds were recorded after 16–18 h of incubation; the interpretation of the results was performed using a photometer. *S. aureus* ATCC 25923 and *E. coli* ATCC 25922 were tested as control strains. All experiments were performed in triplicate.

### 3.5. Screening for and Semi-Quantitative of QS-Inhibitory Activity Using Disk Diffusion Method

The screening and quantification of the QS-inhibitory activity of the tested compounds were performed using the disk diffusion method, the detailed description and optimization of these methods were previously described [71,101,121]. Screening for the QS-inhibitory activity of the tested compounds was performed using the *cross-inoculation method* (see Supplementary Material Figure S2). Briefly, overnight bacterial cultures of *C. violaceum* wt85 and *S. marcescens* AS-1 (at OD_580_ ~ 0.5) grown in LB-B broth were inoculated directly onto LB*-A agar surface in a crossing pattern (see Supplementary Figure S2). Filter paper disks (7.0 mm in diameter, Whatman 3MM) were impregnated with 10 µL of the solutions of the tested compounds (at 1.56–50 µg/mL, depending on the MICs of the respective drugs, see Section 3.4), which were placed at the center of the crossing pattern right after the plates were inoculated. Before the evaluation, the plates were incubated for 48 h at room temperature.

**Figure 5:**
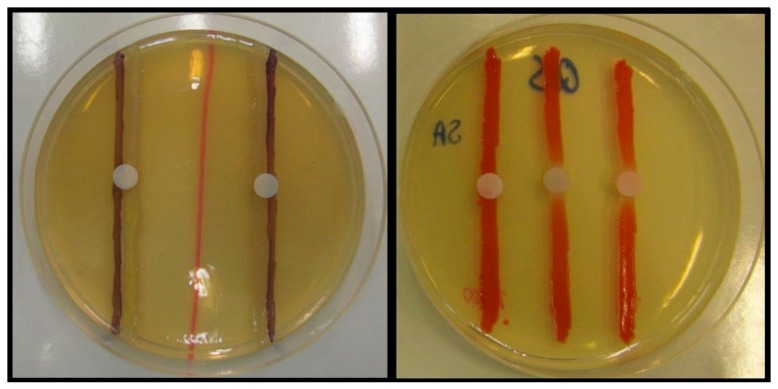
Semi-quantitative QS-inhibition assay, using parallel inoculation disk diffusion method, using *C. violaceum* CV026, and *E. cloacae* 31298 (left), and *S. marcescens* AS-1 (right).

**Figure 6:**
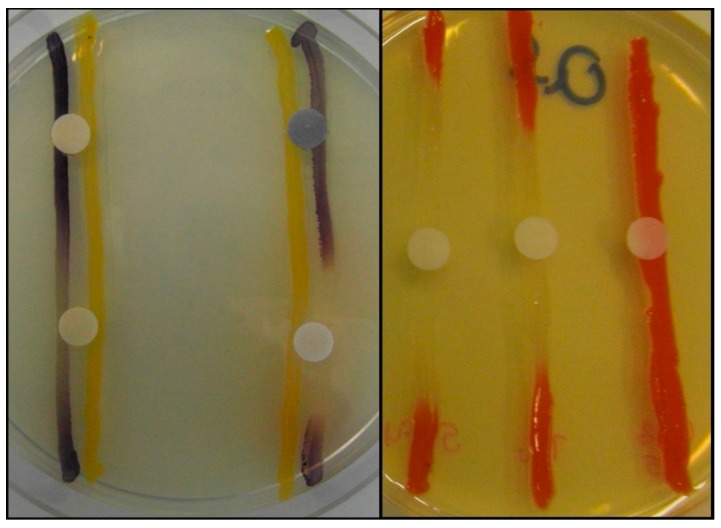
Presentation of positive and negative controls during semi-quantitative QS-inhibition assay, using parallel inoculation disk diffusion method, **Left**: *C. violaceum* CV026 and *Sphingomonas paucimobilis Ezf 10–17* (one effective compounds can be observed in the lower right corner); **Right**: *S. marcescens* AS-1 (two effective compound can be observed on the left side and center).Activity is represented as discolored bacterial colonies with no detected growth inhibition.

If detectable QS-inhibition (discolored, but intact bacterial colonies around the treated paper disk) was observed for a tested pharmaceutical compound, their QS-inhibitory activity was quantified using the *parallel inoculation method* (Figure 5 and Figure 6). Pair combinations of the used sensor strain *C. violaceum* CV026 and the AHL-signal-producing strains (either *E. cloacae* 31298, *S. paucimobilis* Ezf 10–17 or *Novosphingobium* spp. Rr 2–17) were inoculated directly onto the LB*-A agar surface in parallel, at a 5 mm distance from each other, while *S. marcescens* AS-1 was inoculated as a single line (capable of producing prodigiosin from endogenous AHL-signals) [71,101,121]. Filter paper disks (impregnated with 10 µL of the solutions of the different solutions of the tested compounds) were placed on the center of the inoculated line(s). To quantify the QS inhibitory effect of the drugs, the diameter of the QS-inhibition zones (i.e., the size of discolored bacterial colonies with no growth inhibition) around the disks was measured using a caliper. The results of the studies are derived from the average of at least five independent experiments.

## 4. Conclusions

Infections caused by MDROs are associated with excess morbidity (sequelae, decrease in the quality of life) and mortality worldwide. Since the 21st century, antibacterial drug development has been slow to keep up with the rapid developments in the levels of bacterial resistance. Anti-virulence may offer a new wave of potential antibacterial therapeutics in the future, which drugs that will presumably have longer periods of clinical usefulness, compared to antibiotics. QS-based modulation of bacterial virulence is a straightforward and attractive drug development avenue. Nowadays, several thousands of drug compounds are marketed for human therapeutic purposes: these pharmaceuticals may be considered as a potentially untapped source of QS-inhibitory agents with different chemical structures and mechanisms of action, as the pharmacokinetic parameters and tolerability of these compounds have already been demonstrated in vivo. In our experiments, we have demonstrated the antibacterial and QS-inhibitory effects of various pharmaceutical molecules, contributing to the ‘*chemical information space*’ of QS-inhibition. Based on our promising results, further experiments involving the screening of additional pharmaceutical compounds and the utilization of more model systems are warranted.

## Figures and Tables

**Table 1:** Antibacterial activity of tested pharmacological agents (minimal inhibitory concentration values; (µg/mL)) *.

Compounds	*C. violaceum* wt85	*C. violaceum* CV026	*E. cloacae* 31298	*S. paucimobilis* Ezf 10-17	*Novosphingobium* Spp. Rr 2-17	*S. marcescens* AS-1	*E. coli* ATCC 25922	*S. aureus* ATCC 25923
***celecoxib***	>250	>250	>250	>250	>250	>250	>250	**15.6**
***mebendazole***	**62.5**	**62.5**	**125**	**62.5**	**62.5**	**31.3**	**125**	**62.5**
***ivermectin***	>250	>250	>250	>250	>250	>250	>250	**31.3**
***verapamil***	>250	>250	>250	**250**	**250**	>250	>250	**250**
***promethazine***	**31.3**	**31.3**	>250	>250	**250**	**125**	>250	>250
***chlorpromazine***	**15.6**	**15.6**	**125**	**62.5**	**62.5**	**62.5**	>250	>250
***thioridazine***	**15.6**	**15.6**	**62.5**	**31.3**	**31.3**	**15.6**	**31.3**	**125**
***methotrexate***	**125**	**125**	>250	**125**	**125**	>250	>250	>250
***doxorubicin***	**125**	**125**	**250**	**250**	**250**	**125**	**125**	**62.5**
***bleomycin***	**125**	**125**	**125**	**250**	**250**	>250	**125**	**62.5**
***atorvastatin***	**125**	**125**	**31.3**	>250	>250	**125**	>250	**62.5**
***simvastatin***	**250**	**250**	**62.5**	>250	>250	**125**	>250	**125**
***clotrimazole***	>250	>250	>250	>250	>250	>250	>250	**125**
***fluconazole***	>250	>250	>250	>250	>250	>250	>250	**62.5**
***DMSO***	>2 V/V%	>2 V/V%	> 2 V/V%	>2 V/V%	>2 V/V%	>2 V/V%	>2 V/V%	>2 V/V%

* Pharmacological agents not presented in this table had an MIC > 250 µg/mL for all tested bacterial strains.

**Table 2:** Pharmacological agents and food-derived compounds tested for their antibacterial and quorum sensing-inhibitory activities.

*Pharmacological Agents*	*Food-Derived Compounds*
acetyl-salicylic acid [84]	pepper [85]
antifungal azoles [67]	curcumin [86]
auranofin [87]	horse raddish [88]
azathioprine [89]	flavonoids [90]
bithionol [91]	zeaxantin [92]
catecholamines [93]	cranberry juice [94]
celecoxib [69]	resveratrol [95]
coumarines [37,96]	betulinic acid [97]
chloroxazone [57]	ajoene [98]
daunorubicin [99]	essential oils [71,94,100]
diflunisal [101]	
finasteride [102]	
floxuridine [103]	
glyceryl-trinitrate [104]	
ibuprofen [105]	
ivermectin [68]	
local anesthetics [106,107]	
general anesthetics [106,107]	
metformin [108]	
miltefosine [109]	
niclosamide [110]	
parthenolide [111]	
toremifene [112]	
statins [65,66]	
streptozotocin [103]	
Vitamin A [113]	
Vitamin C [114]	
Vitamin D [113,115,116]	
Vitamin K [117]	

## Supplementary Materials

The following are available online at https://www.mdpi.com/2079-6382/8/4/270/s1. Figure S1: Results of a literature search in the PubMed/MEDLINE database on the keywords “quorum sensing” and “quorum quenching”, Figure S2: Screening for QS-inhibitory activity with the cross-inoculation disk diffusion method, using Chromobacterium violaceum wt85 (left) and) and Serratia marcescens AS-1 (right), Table S1: Quorum sensing-inhibitory activity of selected pharmacological agents.
