# Supplementary material for: The Role of Drug Repurposing in the Development of Novel Antimicrobial Drugs: Non-Antibiotic Pharmacological Agents as Quorum Sensing-Inhibitors
Source: Antibiotics (Basel). 2019 Dec 17;8(4):270. doi: 10.3390/antibiotics8040270 (PMC6963710; doi:10.3390/antibiotics8040270)
Supplement: Supplementary file 1 [file antibiotics-08-00270-s001.pdf]

Supplementary material

Communication

The role of drug repurposing in the development of novel antimicrobial drugs: Non-antibiotic pharmacological agents as quorum sensing-inhibitors

Márió Gajdács [1,2]*, Gabriella Spengler [2]

[1] Department of Medical Microbiology and Immunobiology, Faculty of Medicine, University of Szeged; 6720 Szeged, Dóm tér 10., Hungary
[2] Department of Pharmacodynamics and Biopharmacy, Faculty of Pharmacy, University of Szeged, 6720 Szeged, Eötvös utca 6., Hungary 2; spengler.gabriella@med.u-szeged.hu (G.S.)
* Correspondence: gajdacs.mario@pharm.u-szeged.hu; Tel.: +36-62-34-28-43 (M.G.)

Supplementary Figure S1

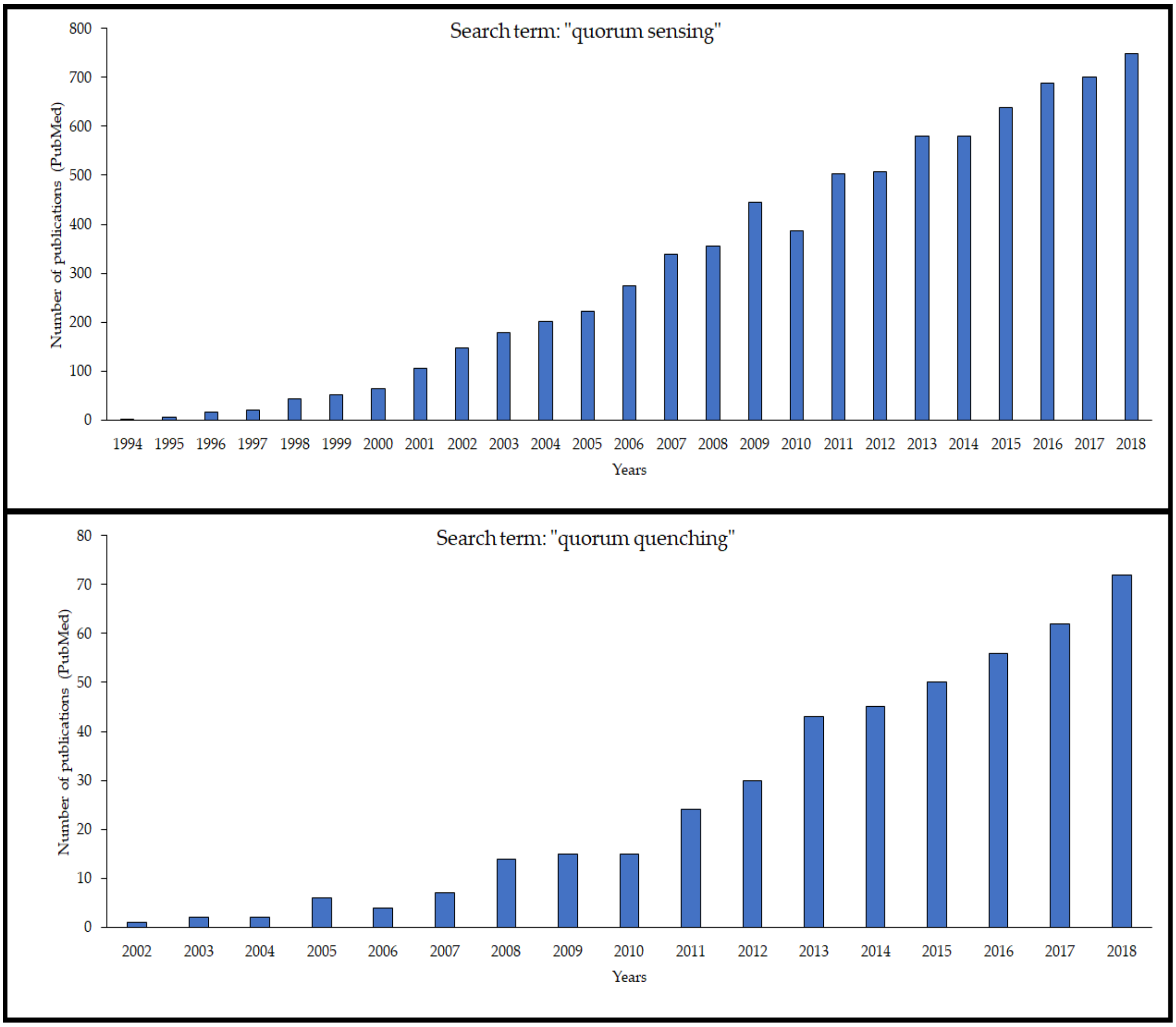

Figure 1. Results of a literature search in the PubMed/MEDLINE database on the keywords "quorum sensing" and "quorum quenching"

Supplementary material

Description: compared to the Year 2000 (n=64), the number of publications in the PubMed/MEDLINE database with the keywords '*quorum sensing*' has increased 7-fold by 2009 (n=445), and 12-fold by 2018 (n=748), respectively, demonstrating the significant interest in quorum sensing-based research. A similar upwards trajectory may be observed for the keyword '*quorum quenching*' **(Figure 1.).**

Supplementary Table S1

Table 1. Quorum sensing-inhibitory activity of selected pharmacological agents
(QS-inhibition zone diameter in mm ± SD)

	**Dose (ng/disk)**	***C. violaceum* CV026 and *E. cloacae* 31298**	***C. violaceum* CV026 and *S. paucimobilis* Ezf 10-17**	***C. violaceum* CV026 and *Novosphingobium* spp. Rr 2-17**	***S. marcescens* AS-1**
acridine orange (AO) (positive control)	1000	38.8 ± 0.9	35.1 ± 1.2	26.3 ± 1.7	46.4 ± 2.1
	500	24.9 ± 1.8	30.0 ± 1.4	19.5 ± 2.2	36.1 ± 1.4
	250	14.6 ± 1.2	16.3 ± 0.9	13.7 ± 1.0	19.2 ± 0.8
	125	-	-	-	-
5-fluorouracil	1000	**68.3 ± 2.4**	**68.0 ± 4.5**	**52.8 ± 1.9**	**71.0 ± 3.3**
	500	**49.5 ± 1.6**	**51.3 ± 2.8**	**31.4 ± 2.2**	**52.0 ± 1.6**
	250	12.0 ± 0.8	**26.2 ± 1.2**	10.2 ± 1.6	**37.2 ± 0.8**
	125	-	4.9 ± 0.2	-	**14.4 ± 0.6**
	62.5	-	-	-	**4.0 ± 0.8**
metamizol-sodium	1000	12.4 ± 1.0	12.6 ± 0.8	9.4 ± 0.2	18.8 ± 0.9
	500	7.2 ± 0.2	7.9 ± 0.6	6.3 ± 0.8	11.0 ± 1.2
	250	-	-	-	-
	125	-	-	-	-
cisplatin	1000	11.1 ± 0.6	10.4 ± 0.9	7.6 ± 0.5	23.9 ± 1.2
	500	6.0 ± 0.4	7.1 ± 0.9	5.1 ± 0.3	18.8 ± 0.3
	250	-	-	-	5.1 ± 0.3
	125	-	-	-	-
methotrexate	1000	14.0 ± 0.6	16.1 ± 1.0	12.8 ± 0.6	19.3 ± 0.7
	500	11.6 ± 1.2	13.8 ± 0.5	10.1 ± 0.3	16.5 ± 1.7
	250	-	4.6 ± 0.4	-	5.9 ± 0.5
	125	-	-	-	-
bleomycin	500	**40.0 ± 3.1**	**37.2 ± 1.8**	**29.0 ± 1.7**	**46.9 ± 0.9**
	250	**27.7 ± 2.4**	**30.2 ± 1.4**	**21.4 ± 0.8**	**33.3 ± 0.8**
	125	13.0 ± 1.8	8.2 ± 2.1	9.0 ± 0.5	17.4 ± 1.2
	62.5	3.0 ± 0.5	-	-	6.6 ± 0.9
	500	**40.2 ± 2.4**	**38.9 ± 1.6**	**27.1± 2.4**	**16.8 ± 0.9**

Supplementary material

	250	**31.0 ± 4.6**	**29.7 ± 0.7**	**22.8 ± 1.3**	**12.2 ± 1.2**
promethazine	125	**7.8 ± 1.0**	**17.0 ± 1.0**	**6.6 ± 0.8**	**3.9 ± 0.6**
	62.5	-	**4.2 ± 0.5**	-	-
	250	**42.1 ± 1.8**	**46.1 ± 2.4**	**36.5 ± 1.9**	**26.0 ± 1.8**
	125	**33.7 ± 1.5**	**40.3 ± 2.5**	**31.1 ± 2.0**	**19.8 ± 1.2**
chlorpromazine	62.5	**14.8 ± 0.6**	**22.8 ± 0.8**	**11.2 ± 0.7**	**12.0 ± 0.4**
	31.2	**7.1 ± 0.3**	**6.9 ± 1.0**	-	**4.5 ± 0.6**
	250	**46.3 ± 1.4**	**45.1 ± 2.6**	**40.0 ± 1.7**	**49.1 ± 1.6**
	125	**38.8 ± 0.3**	**36.7 ± 1.6**	**34.0 ± 0.9**	**40.0 ± 1.9**
thioridazine	62.5	**13.2 ± 1.2**	**24.0 ± 0.3**	**16.4 ± 0.7**	**21.3 ± 0.7**
	31.2	**6.0 ± 1.0**	**10.5 ± 0.7**	**5.5 ± 1.0**	**9.0 ± 1.0**

Doses in italics represent doses higher than the MIC values of the respective compounds

Values in boldface represent QS-inhibitory activity, which is more potent than the activity of the positive control

Supplementary Figure S2

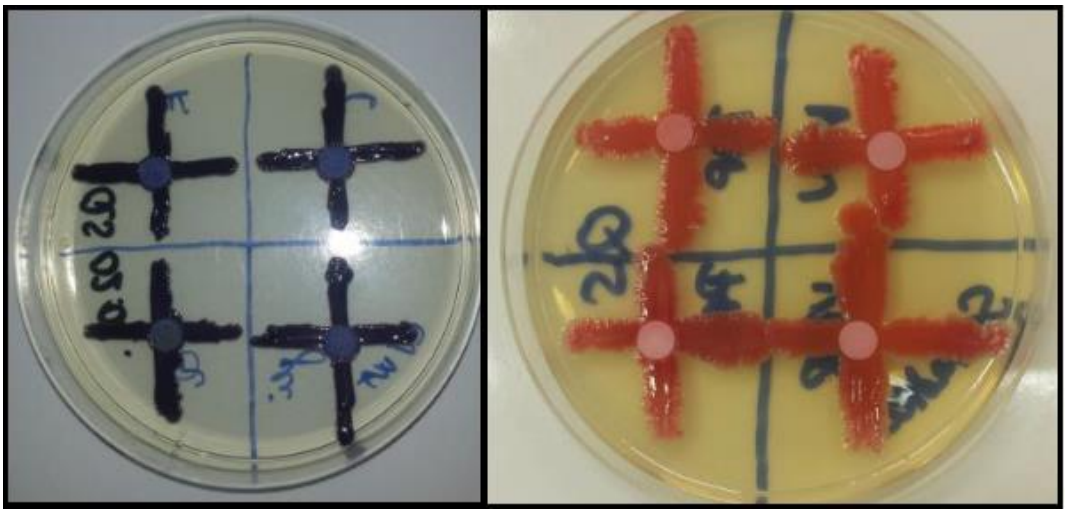

Figure 2. Screening for QS-inhibitory activity with the cross-inoculation disk diffusion method, using *Chromobacterium violaceum* wt85 (left) and) and *Serratia marcescens* AS-1 (right)
